# P-468. Prevalence and Associated Factors of Patients Presenting with Advanced HIV Disease in Tertiary Hospital in Bangkok

**DOI:** 10.1093/ofid/ofae631.667

**Published:** 2025-01-29

**Authors:** Artima Songaurailam, Suppavit Chumsantivut, Pattraporn Piyapan

**Affiliations:** Faculty of Medicine, Chulalongkorn University, Krung Thep, Thailand; Faculty of Medicine, Chulalongkorn University, Krung Thep, Thailand; Bhumibol Adulyadej Hospital, Bangkok, Krung Thep, Thailand

## Abstract

**Background:**

Late entry to healthcare services for HIV patients leads to adverse outcomes and increased mortality particularly advanced HIV disease (AHD) who first presented with opportunistic infection (OI). However, epidemiological data on AHD among Thai patients in suburban government hospital during the Treat-All era was lacking and required to guide the healthcare strategies. This study aims to assess the prevalence, clinical characteristics and risk factors of patients presenting with AHD at Bhumibol Adulyadej Hospital, a tertiary hospital in Bangkok.

**Figure:**
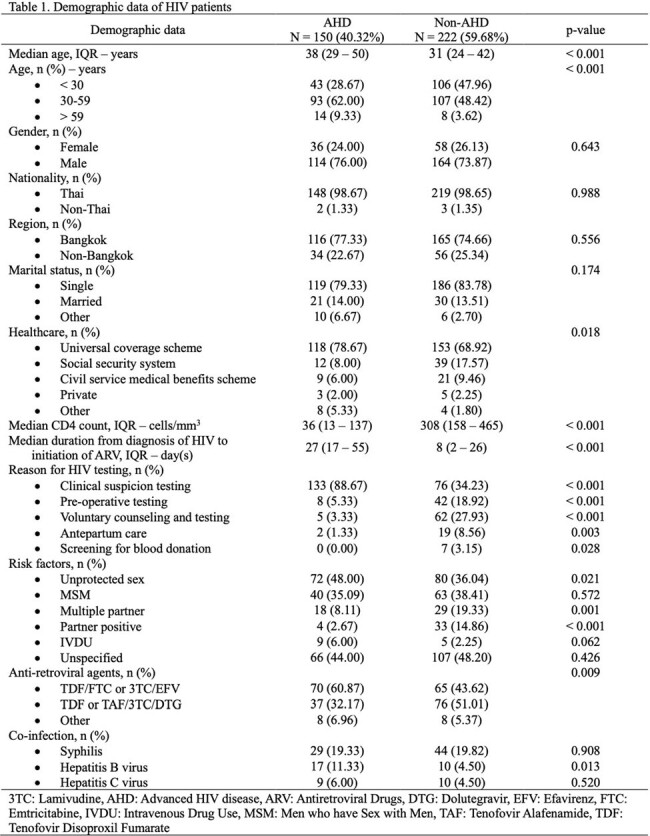

**Methods:**

A retrospective cross-sectional study was conducted among newly diagnosed adult HIV patients from 1 October 2019 to 30 September 2023, through electronic chart review. The WHO clinical staging 3-4 was used to define AHD. Logistic regression was used to identify factors associated with AHD.

**Figure:**
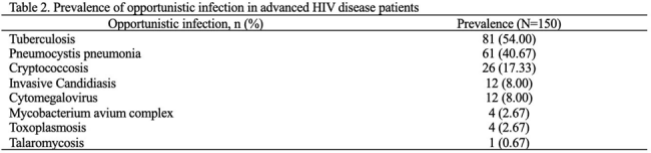

**Results:**

Among 372 patients, 40.32% presented with AHD, with a median CD4 cell count of 36 (IQR 13-137) cells/mm^3^. Most of the newly diagnosed HIV patients were single males residing in the hospital’s service area. AHD patients were predominantly aged 30-59 years (62.00%) and identified through clinical suspicious testing (88.67%) rather than voluntary counseling testing (VCT). Risk factors of AHD included ages 30-59 years (adjusted OR (aOR) 1.94 [95%CI 1.13-3.34]) and > 59 years (aOR 4.58 [95%CI 1.49-14.06]), clinical suspicious testing (aOR 11.98 [95%CI 6.61-21.71]), unprotected sex (aOR 1.74 [95%CI 1.01-2.98]), and partner seropositive (aOR 0.18 [95%CI 0.05-0.62]). One-year mortality was significantly higher in AHD who were diagnosed from 2019-2022 (20.31% V.S. 9.88%, p=0.011). The most common OIs among AHD were tuberculosis (54.00%), pneumocystis pneumonia (40.67%) and cryptococcosis (17.33%) consecutively.

**Figure:**
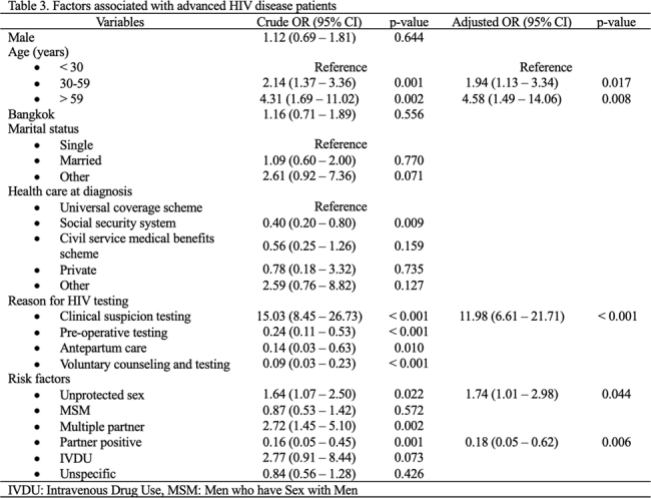

**Conclusion:**

Presenting with AHD was common and associated with high mortality. VCT plays a minor role in the study population. The early HIV infection detection program remains needed in all risk groups but should target heterosexual males aged > 30.

**Disclosures:**

**All Authors**: No reported disclosures

